# Systemic Inflammatory Mediators Are Effective Biomarkers for Predicting Adverse Outcomes in Clostridioides difficile Infection

**DOI:** 10.1128/mBio.00180-20

**Published:** 2020-05-05

**Authors:** Michael G. Dieterle, Rosemary Putler, D. Alexander Perry, Anitha Menon, Lisa Abernathy-Close, Naomi S. Perlman, Aline Penkevich, Alex Standke, Micah Keidan, Kimberly C. Vendrov, Ingrid L. Bergin, Vincent B. Young, Krishna Rao

**Affiliations:** a Medical Scientist Training Program, University of Michigan Medical School, Ann Arbor, Michigan, USA; b Department of Microbiology and Immunology, University of Michigan, Ann Arbor, Michigan, USA; c Infectious Diseases Division, Department of Internal Medicine, University of Michigan, Ann Arbor, Michigan, USA; d Unit for Laboratory Animal Medicine, University of Michigan, Ann Arbor, Michigan, USA; University of Oklahoma Health Sciences Center

**Keywords:** biomarkers, *Clostridioides difficile*, *Clostridium difficile*, machine learning, predictive modeling, cytokines

## Abstract

Clostridioides difficile infection (CDI) can result in severe disease and death, with no accurate models that allow for early prediction of adverse outcomes. To address this need, we sought to develop serum-based biomarker models to predict CDI outcomes. We prospectively collected sera ≤48 h after diagnosis of CDI in two cohorts. Biomarkers were measured with a custom multiplex bead array assay. Patients were classified using IDSA severity criteria and the development of disease-related complications (DRCs), which were defined as ICU admission, colectomy, and/or death attributed to CDI. Unadjusted and adjusted models were built using logistic and elastic net modeling. The best model for severity included procalcitonin (PCT) and hepatocyte growth factor (HGF) with an area (AUC) under the receiver operating characteristic (ROC) curve of 0.74 (95% confidence interval, 0.67 to 0.81). The best model for 30-day mortality included interleukin-8 (IL-8), PCT, CXCL-5, IP-10, and IL-2Rα with an AUC of 0.89 (0.84 to 0.95). The best model for DRCs included IL-8, procalcitonin, HGF, and IL-2Rα with an AUC of 0.84 (0.73 to 0.94). To validate our models, we employed experimental infection of mice with C. difficile. Antibiotic-treated mice were challenged with C. difficile and a similar panel of serum biomarkers was measured. Applying each model to the mouse cohort of severe and nonsevere CDI revealed AUCs of 0.59 (0.44 to 0.74), 0.96 (0.90 to 1.0), and 0.89 (0.81 to 0.97). In both human and murine CDI, models based on serum biomarkers predicted adverse CDI outcomes. Our results support the use of serum-based biomarker panels to inform Clostridioides difficile infection treatment.

## Abstract (precis)

Each year in the United States, Clostridioides difficile causes nearly 500,000 gastrointestinal infections that range from mild diarrhea to severe colitis and death. The ability to identify patients at increased risk for severe disease or mortality at the time of diagnosis of C. difficile infection (CDI) would allow clinicians to effectively allocate disease modifying therapies. In this study, we developed models consisting of only a small number of serum biomarkers that are capable of predicting both 30-day all-cause mortality and adverse outcomes of patients at time of CDI diagnosis. We were able to validate these models through experimental mouse infection. This provides evidence that the biomarkers reflect the underlying pathophysiology and that our mouse model of CDI reflects the pathogenesis of human infection. Predictive models can not only assist clinicians in identifying patients at risk for severe CDI but also be utilized for targeted enrollment in clinical trials aimed at reduction of adverse outcomes from severe CDI.

## INTRODUCTION

Clostridioides difficile is a spore-forming bacillus that causes nearly 500,000 cases of toxin-mediated gastrointestinal illness yearly in the United States, with 29,300 deaths and a cost of 1.5 billion dollars annually (1). The pathogenesis of C. difficile infection (CDI) involves local toxin production within the intestines, leading to diarrhea and intestinal wall inflammation. Some patients experience severe colitis, along with a systemic inflammatory response as previously characterized (2).

We currently lack highly accurate predictive tools to assist with clinical decisions following CDI diagnosis. The development of accurate predictive models for adverse outcomes could guide the use of emerging treatments for CDI that can ameliorate or prevent disease-related complications (DRCs) such as ICU admission, colectomy, or death (3). For instance, fidaxomicin is costlier than vancomycin, while fecal transplants carry the risk of alterations to the host microbiome with unknown long-term effects, as well as transmission of enteric pathogens (4). Widespread deployment of these novel treatments in patients with CDI is impractical due to expense, invasiveness, and undetermined safety profiles, necessitating the development of tools for patient risk stratification treatment selection optimization.

The Infectious Diseases Society of America (IDSA) and Society for Healthcare Epidemiology of America (SHEA) guidelines use measurements of systemic immune response (white blood cell [WBC] count > 15,000) or signs of renal dysfunction (creatinine > 1.5) to define severe CDI (5). Further signs of organ failure (shock, hypotension, ileus, or megacolon) are used to define complicated CDI. While this classification system guides management decisions, the features used are late findings and do not always allow for early identification of high-risk individuals. For instance, in a study of two cohorts consisting of 156 and 272 unique CDI cases, of the 23 all-cause mortality cases, 10 of the patients (43.5%) did not meet IDSA severity criterion at time of diagnosis. An ideal model would identify cases of CDI at the time of diagnosis that are progressing toward severe systemic disease, so that treatments to halt disease progression can be started. Models built from baseline clinical variables or standard laboratory measurements have met with limited success in accurately predicting adverse outcomes, or they do not validate externally (6–12). Therefore, we set out to determine if predictive models built from a panel of multiple inflammatory mediators measured at diagnosis of CDI can accurately predict adverse outcomes, specifically, 30-day all-cause mortality and DRCs defined as ICU admission, colectomy, and/or death attributed to CDI. To validate these findings and provide further evidence of the utility of mouse models for CDI, we employed an experimental C. difficile infection in mice and tested the capability of the biomarker-based model to determine high disease severity in these mice.

## RESULTS

### Serum markers of epithelial damage, inflammation, and neutrophilic migration are significantly associated with mortality and disease-related complications.

We studied an initial pilot cohort of 156 patients with CDI, of whom 58 (37.2%) met IDSA severity criteria, 4 (2.6%) died within 30 days, and 10 (6.4%) had disease-related complications. Of the 4 patients with CDI who died within 30 days, 2 did not meet IDSA severity criteria at the time of diagnosis. Serum collected near time of diagnosis was tested with a custom panel for serum biomarkers ranging from inflammatory markers to epithelial growth factors (Table 1). Biomarker profiles of serum from severe and nonsevere cases showed separation by principal-component analysis (see Fig. S1A and B in the supplemental material), while redundancy analysis (RDA) of biomarkers differentiated severe and nonsevere episodes by permutational multivariate analysis of variance (MANOVA) (*P = *0.005) and differentiated cases that developed DRCs (*P = *0.025) (Fig. S1C and D). These biomarkers did not distinguish between patients who died within 30 days of diagnosis, most likely due to the limited number of 30-day mortality cases in our pilot study (*n* = 4). Unadjusted logistic regression revealed that interleukin-6 (IL-6), procalcitonin (PCT), IL-8, IL-2Rα, and hepatocyte growth factor (HGF) were significantly associated with severity (*P *values of <0.001, <0.01, <0.05, <0.05, and <0.05, respectively). All of these biomarkers except procalcitonin were also significantly associated with DRCs but not overall 30-day mortality (Table S1).

**TABLE 1:** Support for inclusion of inflammatory mediators previously shown to be associated with CDI severity and adverse outcomes^*a*^

Inflammatory mediator (abbreviation)	Alternative name(s) and/or abbreviation(s)	Prior studies in CDI/UC^*b*^
Tumor necrosis factor alpha (TNF-α)		Olson et al. (21), Brito et al. (22), Klapproth and Sasaki (23)
Interleukin-2 receptor α (IL-2Rα)	CD25	Rao et al. (2)
Interleukin-4 (IL-4)		Connelly et al. (24)
Interleukin-6 (IL-6)		Rao et al. (2)
Interleukin-8 (IL-8)	Neutrophil chemotactic factor	Rao et al. (2), Steiner et al. (25), Jiang et al. (26)
Interleukin-15 (IL-15)		Rao et al. (2)
Interleukin-22 (IL-22)		Sadighi Akha et al. (27)
Interleukin-23 (IL-23)		Cowardin et al. (28), Buonomo et al. (29)
Chemokine (C-C motif) ligand 2 (CCL2)	Monocyte chemotactic protein 1 (MCP-1) or small inducible cytokine A2 (SCYA2)	Rao et al. (2)
CCL5	RANTES	Rao et al. (2)
Chemokine (C-C motif) ligand 4 (CCL4)	Macrophage inflammatory protein 1β (MIP-1β)	Rao et al. (2)
Chemokine (C-X-C motif) ligand 5 (CXCL5)		El Feghaly et al. (30)
Chemokine (C-X-C motif) ligand 9 (CXCL9)	Monokine induced by gamma interferon (MIG)	Rao et al. (2)
Hepatocyte growth factor (HGF)		Rao et al. (2).
Epidermal growth factor (EGF)		Rao et al. (2)
Chemokine (C-X-C motif) ligand 10 (CXCL10)	Interferon gamma-induced protein 10 (IP-10) or small inducible cytokine B10 (SCYB10)	Rao et al. (2)
Procalcitonin (PCT)		Rao et al. (31)

a Reproduced with permission from the work of Limsrivilai et al. (32).

b UC, ulcerative colitis.

10.1128/mBio.00180-20.1 TABLE S1 Pilot cohort: biomarker population statistics and simple unadjusted logistic regression analysis for Infectious Diseases Society of America (IDSA) severity and the disease outcomes (30-day all-cause mortality and disease-related complications [DRCs]). Download Table S1, DOCX file, 0.02 MB. Copyright © 2020 Dieterle et al. 2020 Dieterle et al. This content is distributed under the terms of the Creative Commons Attribution 4.0 International license.

10.1128/mBio.00180-20.4 FIG S1 Pilot cohort analysis: principal-component analysis (PCA) and redundancy analysis (RDA) for predicting adverse outcomes with inflammatory mediators. (A) Biplot showing the direction of each inflammatory mediator with respect to PCA axes 1 and 2 in the pilot cohort. Of note, interleukin-6 (IL-6), IL-2R, and hepatocyte growth factor (HGF) are strong drivers in the same direction as the separation in the PCA plot colored by IDSA severity (B). PCA shows separation between severe (1) and nonsevere (0) CDI cases, with PC1 explaining 30% of the variance and PC2 explaining 23.3% of the variance. (C and D) RDA axis 1 versus PC1 plots for IDSA severity (C) and DRCs (D). RDA differentiated the biomarker profile of the positive (1) and negative (0) cases for IDSA severity (C) and DRCs (D) by permutational multivariate analysis of variance (PERMANOVA) (*P* < 0.01 and *P* < 0.05, respectively). Download FIG S1, EPS file, 2.1 MB. Copyright © 2020 Dieterle et al. 2020 Dieterle et al. This content is distributed under the terms of the Creative Commons Attribution 4.0 International license.

We employed a validation cohort of 272 unique CDI cases among 253 patients, of whom 71 (26.1%) met IDSA severity criteria, 19 (7.0%) died within 30 days, and 18 (6.6%) had DRCs (Table 2). Eight of 19 patients experiencing 30-day all-cause mortality did not meet IDSA severity criteria at the time of diagnosis. There were 14 patients that experienced 30-day all-cause mortality and developed DRCs. Similar to the case with the pilot, biomarker-based RDA of the validation cohort differentiated severe and nonsevere CDI cases by permutational MANOVA (*P = *0.001) and DRCs (*P = *0.002). With the increase in the number of patients who died, biomarker profiles from patients with 30-day mortality were also differentiated by RDA (*P = *0.001) (Fig. S2). Characterization of biomarker associations with each outcome was performed with unadjusted logistic regression and showed that 12 of the 17 inflammatory markers were individually associated with at least one outcome, with 6 biomarkers (HGF, procalcitonin, IL-6, IL-2Rα, IL-8, and tumor necrosis factor alpha [TNF-α]) significantly associated with all three outcomes. With unadjusted inflammatory mediators, the most significant positive associated biomarkers (*P < *0.001) with IDSA severity were HGF, PCT, IL-6, and IL-2Rα, with 30-day mortality were IL-2Rα, PCT, IL-8, and IP-10, and with DRCs were PCT, IL-8, and IL-2Rα (Table 3). All associations are shown in Table S2. These findings validate the associations between biomarkers and adverse outcomes seen in the pilot cohort.

**TABLE 2:** Cohort demographics and pertinent patient information

Demographic category	Subcategory	Value for:	
		Pilot	Validation
No. of cases		156	272
Age (yrs)		56 ± 18	55 ± 21
Sex	Male	67 (43.0%)	131 (48.2%)
	Female	89 (57.0%)	141 (51.8%)
Race	Caucasian	137 (87.8%)	236 (86.8%)
	Black or African American	10 (6.4%)	18 (6.6%)
	Asian	0 (0%)	4 (1.5%)
	American Indian or Alaska Native	2 (1.3%)	3 (1.1%)
	Native Hawaiian and Pacific Islander	1 (0.6%)	0 (0%)
	Other or unknown	6 (3.9%)	11 (4.0%)
Ribotypes	027 ribotype	15 (9.6%)	25 (9.2%)
	014-020 ribotype	30 (19.2%)	47 (17.3%)
Method of CDI diagnosis	Toxins A/B enzyme immunoassay	71 (46%)	69 (25%)
	Reflex to PCR for *tcdB* gene	85 (54%)	203 (75%)
Disease measures	IDSA severity	58 (37.2%)	71 (26.1%)
	30-day mortality	4 (2.6%)	19 (7.0%)
	DRCs	10 (6.4%)	18 (6.6%)
	Subset with 30-day all-cause mortality and DRCs	2 (1.3%)	14 (5.2%)
Pertinent medical history	Elixhauser score		4.6 ± 3.3
	Concurrent antibiotics	112 (71.8%)	87 (32.0%)
	History of C. difficile infection	40 (25.6%)	52 (19.6%)
	Inflammamatory bowel disease		41 (15.1%)

**TABLE 3:** Validation cohort: top six inflammatory mediators by simple unadjusted logistic regression for IDSA severity, 30-day all-cause mortality, and disease-related complications^*a*^

Unadjusted analysis for IDSA severity				Unadjusted analysis for 30-day all-cause mortality				Unadjusted analysis for DRC			
Biomarker	OR	OR sig.	AUC	Biomarker	OR	OR sig.	AUC	Biomarker	OR	OR sig.	AUC
HGF	1.97 (1.49–2.60)	***	0.71 (0.64–0.78)	IL-2Rα	8.28 (3.41–20.11)	***	0.85 (0.77–0.92)	PCT	1.94 (1.43–2.64)	***	0.82 (0.75–0.90)
PCT	1.57 (1.3–1.89)	***	0.69 (0.62–0.76)	PCT	1.92 (1.42–2.58)	***	0.82 (0.73–0.9)	IL-8	2.03 (1.44–2.86)	***	0.78 (0.67–0.88)
IL-6	1.39 (1.17–1.65)	***	0.68 (0.62–0.75)	IL-8	2.03 (1.45–2.86)	***	0.80 (0.71–0.90)	IL-2Rα	4.86 (2.16–10.94)	***	0.79 (0.69–0.88)
IL-2Rα	2.29 (1.47–3.57)	***	0.65 (0.58–0.72)	IP-10	1.76 (1.31–2.36)	***	0.67 (0.54–0.79)	IL-6	1.49 (1.17–1.90)	**	0.69 (0.54–0.84)
IL-8	1.44 (1.14–1.82)	**	0.64 (0.57–0.71)	EGF	0.59 (0.43–0.8)	***	0.70 (0.58–0.83)	HGF	1.94 (1.3–2.89)	**	0.71 (0.59–0.84)
TNF-α	2.87 (1.29–6.39)	**	0.61| (0.53–0.69)	CXCL-5	0.53 (0.36–0.8)	**	0.75 (0.65–0.85)	IP-10	1.42 (1.05–1.94)	*	0.62 (0.49–0.75)

a OR, odds ratio; sig., significance. *, *P* ≤ 0.05; **, *P* ≤0.01; ***, *P* ≤ 0.001.

10.1128/mBio.00180-20.2 TABLE S2 Validation cohort: biomarker population statistics and simple unadjusted logistic regression for IDSA severity and the disease outcomes (30-day mortality and disease-related complications). Download Table S2, DOCX file, 0.02 MB. Copyright © 2020 Dieterle et al. 2020 Dieterle et al. This content is distributed under the terms of the Creative Commons Attribution 4.0 International license.

10.1128/mBio.00180-20.5 FIG S2 Validation cohort analysis: PCA and RDA for predicting adverse outcomes with inflammatory mediators. (A) Biplot showing the direction of each inflammatory mediator with respect to PCA axes 1 and 2 in the validation cohort. (B) PC1 and PC2 of the validation cohort inflammatory mediator profiles colored by IDSA severity (left), 30-day mortality (center), and DRCs (right), with PC1 explaining 29.1% and PC2 explaining 21.2% of the variance. Cases positive for severity, 30-day mortality, or DRCs are colored in red (1), and those cases that are negative for those outcomes are blue (0). (C) RDA axis 1 versus PC1 plots for IDSA severity (left), 30-day mortality (center), and DRCs (right), all of which RDA differentiated biomarker profiles of positive (red) and negative (blue) cases by PERMANOVA (*P <* 0.001, *P <* 0.001, and *P <* 0.01, respectively). Download FIG S2, EPS file, 2.7 MB. Copyright © 2020 Dieterle et al. 2020 Dieterle et al. This content is distributed under the terms of the Creative Commons Attribution 4.0 International license.

### Development of high-performance, multivariable models to estimate CDI severity and predict adverse outcomes.

While logistic regression models were initially produced to test feasibility of predicting 30-day all-cause mortality and DRCs from serum biomarkers at diagnosis (Table S3), these models are often not useful outside the particular cohort in which they were built. To produce more refined and generalizable models, we used 5-fold cross-validated elastic net regression modeling. As our goal was not to produce necessarily the best model but to describe which biomarkers have the potential to predict adverse outcomes in a generalizable way that would be most likely to validate in external cohorts, we show the modeling results for a range of tuning parameters. Alpha values were tested from pure ridge regression (alpha = 0) to pure lasso regression (alpha = 1), allowing the visualization of which biomarkers are retained in the model as the inclusion criterion becomes more stringent (toward lasso regression). Additionally, biomarker inclusion is impacted by the selection of lambda, where deviance was within 1 standard error of the minimum (1se) or at the minimum (min).

10.1128/mBio.00180-20.3 TABLE S3 Logistic regression with backward selection for estimating IDSA severity and predicting adverse outcomes (30-day mortality and DRCs). The resulting AUC is stable for each outcome across biomarker inclusion and selection processes. mAIC, stepwise regression, backward selection with all biomarkers; m25AIC, stepwise regression, backward selection including only biomarkers with *P* values of <0.25 from unadjusted logistic regression; mLRT, stepwise regression, drop one selection with all biomarkers; m25LRT, stepwise regression, drop one selection including only biomarkers with *P* values of <0.25 from unadjusted logistic regression. Download Table S3, DOCX file, 0.01 MB. Copyright © 2020 Dieterle et al. 2020 Dieterle et al. This content is distributed under the terms of the Creative Commons Attribution 4.0 International license.

**FIG 1:**
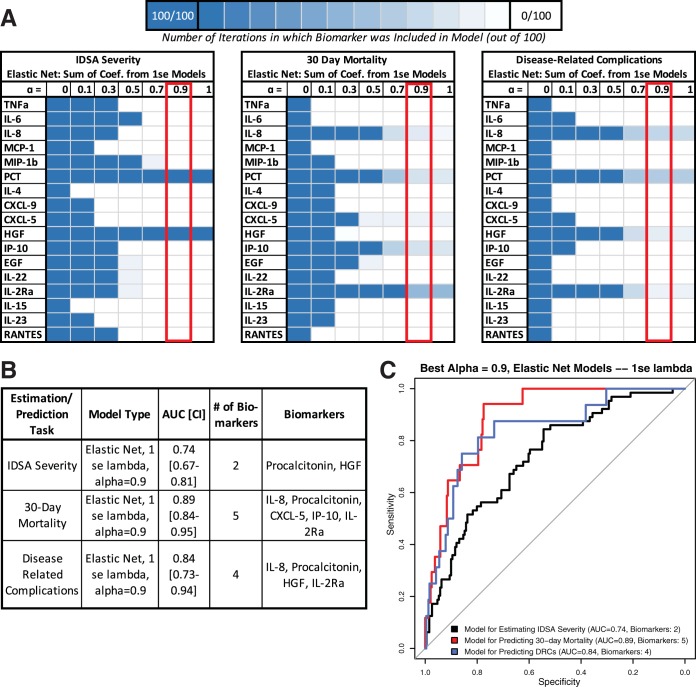
Biomarker inclusion and AUCs for 1 se lambda Glmnet models across 100 iterations for estimating IDSA severity or predicting adverse outcomes. (A) Table showing which biomarkers are included in each Glmnet model. Inclusion was determined by (i) classification task (estimating IDSA severity or predicting adverse outcomes) and (ii) the penalty for including additional low yield variables. Each model was performed across 100 iterations with different initial seeds for each value of alpha. An alpha value closer to 0 weights toward ridge regression, and a value closer to 1 weights toward lasso regression. Lasso regression places a higher penalty on including additional biomarkers, resulting in fewer biomarkers included in the final model for higher alpha values. The color of each square indicates out of the 100 iterations how many times that individual biomarker was included in the produced models for the given alpha value. (B) Table showing the performance of the best model with an alpha value of 0.9 and biomarkers included. (C) ROCs and AUCs for the best models with an alpha value of 0.9.

As smaller models are more useful for clinical applications and performance did not differ drastically between the min (higher potential for overfitting) and 1se (higher potential for being generalizable) models, biomarker inclusion for each model and the area (AUC) under the receiver operating characteristic (ROC) curve performance for the 1se models are shown in Fig. 1, while the results for the min models are shown in Fig. S3. To create the most parsimonious model, the 0.9 models are strongly weighted to reduce unnecessary biomarkers and are the chosen highlighted models, although similar performance is seen across lambda and alpha values. ROCs and AUCs for the best elastic net models at each alpha value are shown in Fig. S4, highlighting the stability of the model performance with decreasing biomarker inclusion.

10.1128/mBio.00180-20.6 FIG S3 Biomarker inclusion and AUCs for 1 min lambda Glmnet models across 100 iterations for estimating IDSA severity or predicting adverse outcomes. (A) Table showing which biomarkers are included in each Glmnet model. Inclusion was determined by (i) classification task (estimating IDSA severity or predicting adverse outcomes) and (ii) the penalty for including additional low-yield variables. Each model was performed across 100 iterations, with different initial seeds for each value of alpha. An alpha value closer to 0 weights towards ridge regression, and a value closer to 1 weights towards lasso regression. Lasso regression places a higher penalty on including additional biomarkers, resulting in fewer biomarkers included in the final model for higher alpha values. The color of each square indicates out of the 100 iterations how many times that individual biomarker was included in the produced models for the given alpha value. (B) Table showing the performance of the best model with an alpha value of 0.9 model and biomarkers included. (C) ROCs and AUCs for the models with an alpha value of 0.9. Download FIG S3, EPS file, 2.5 MB. Copyright © 2020 Dieterle et al. 2020 Dieterle et al. This content is distributed under the terms of the Creative Commons Attribution 4.0 International license.

10.1128/mBio.00180-20.7 FIG S4 Stability of elastic net modeling for predicting adverse CDI outcomes and estimating IDSA severity with adjustment of lambda and alpha values. An alpha value closer to 1 weights towards lasso regression, which increases the penalty of including more variables in the model. Each value of alpha was utilized in 100 model iterations across different initial seeds, with the best model by highest AUC chosen and plotted for 1se lambda models (A) and min lambda (B) for each outcome or estimation task. Minimum lambda models select the minimum value resulting in a more overfit model with increased number of biomarkers, while the 1se lambda models select a value 1 standard deviation away, resulting in a more generalizable model with fewer biomarkers. Comparing the same column in panels A and B, it can be seen that while the AUC was higher in the min lambda models, there was a substantial increase in biomarkers included. Therefore, 1se models with an alpha value of 0.9 retain high AUC performance while limiting biomarker inclusion for improved clinical application. Download FIG S4, EPS file, 2.5 MB. Copyright © 2020 Dieterle et al. 2020 Dieterle et al. This content is distributed under the terms of the Creative Commons Attribution 4.0 International license.

For IDSA severity estimation, elastic net modeling shows that PCT and HGF are included in all models and are the only biomarkers in 1se models with alpha values of >0.5. The 1se (alpha = 0.9) model includes 2 biomarkers and produces an AUC of 0.74 (0.67 to 0.81) (Fig. 1), while the min (alpha = 0.9) model includes 13 biomarkers and produces an AUC of 0.78 (0.71 to 0.84) (Fig. S3).

For 30-day mortality prediction, elastic net modeling shows that IL-8, PCT, IP-10, and IL-2Rα are the most included biomarkers for 1se models and are included in all min models along with CXCL-5. The 1se (alpha = 0.9) model includes 5 biomarkers and produces an AUC of 0.89 (0.84 to 0.95) (Fig. 1), while the min (alpha = 0.9) model includes 12 biomarkers and produces an AUC of 0.91 (0.85 to 0.97) (Fig. S3).

For DRC prediction, elastic net modeling shows that IL-8, PCT, HGF, and IL-2Rα were included in most 1se models and all min models. The 1se (alpha = 0.9) model includes 4 biomarkers and produces an AUC of 0.84 (0.73 to 0.94) (Fig. 1), while the min (alpha = 0.9) model includes 4 biomarkers and produces an AUC of 0.85 (0.74 to 0.96) (Fig. S3). The same biomarkers were included in both models, indicating that determining DRCs is highly dependent on these four markers.

Regardless of model parameters, performances were similar across the largest and smallest models for each outcome and had AUCs higher than the highest among individual biomarker regression models. PCT was the only shared biomarker between 30-day mortality and IDSA severity models. DRC models included the two most significant biomarkers for IDSA severity (HGF and PCT) as well as two others found in 30-day mortality models (IL-2Rα and IL-8). This indicates that the task of predicting DRCs has a solution that overlaps at least in part with estimating IDSA severity and predicting 30-day mortality. Similar to results from logistic regression modeling, the best-performing models were for 30-day mortality, followed closely by DRC, and the worst performance was seen in models for estimating IDSA severity.

### Biomarker-based models outperform basic clinical models for predicting 30-day mortality and DRCs.

**FIG 2:**
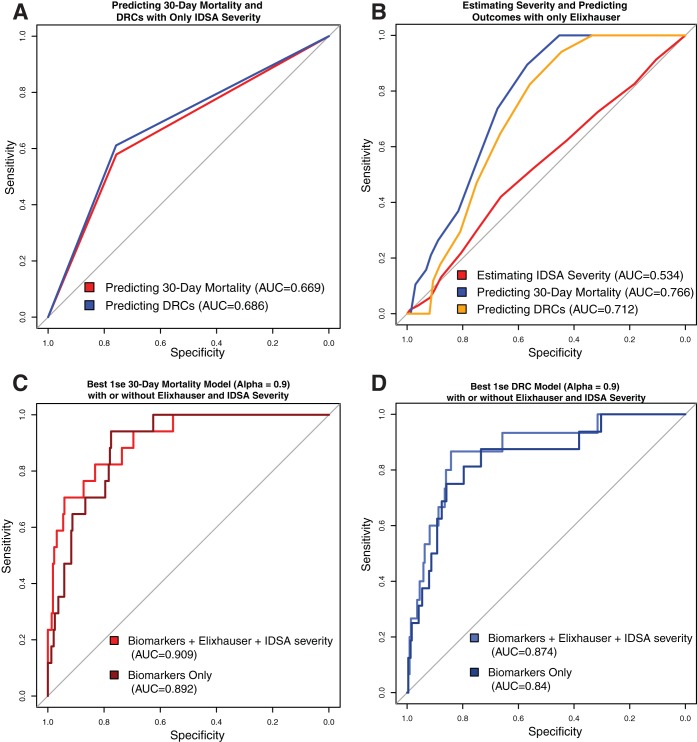
IDSA severity and Elixhauser perform worse than biomarker models, but slightly improve performance when added to biomarker models directly. (A) ROCs and AUCs for logistic regression using only IDSA severity to predict 30-day mortality and DRCs. (B) ROCs and AUCs for best 1se models using only Elixhauser score to predict 30-day mortality, DRCs, and IDSA severity. (C and D) ROCs and AUCs for predicting 30-day mortality and DRCs with best 1se elastic net biomarker model alone or with Elixhauser score and IDSA severity.

IDSA severity is used clinically to assess the severity of CDI and inform treatment, while the Elixhauser comorbidity index (Elixhauser), which was developed in order to predict mortality, is used as an aggregate measure of the burden of comorbid disease at baseline. We used IDSA severity and Elixhauser to estimate adverse outcomes and compare to our biomarker-based models. Simple logistic regression models showed that IDSA severity was significantly associated with 30-day all-cause mortality (*P = *0.003; AUC= 0.67 [0.55 to 0.79]) and DRCs (*P = *0.002; AUC= 0.69 [0.57 to 0.80]) but performed substantially worse than our biomarker models (Fig. 2A). Simple logistic regression models showed that Elixhauser index was significantly associated with 30-day all-cause mortality (*P < *0.001; AUC= 0.77 [0.69 to 0.84]) and DRCs (*P = *0.018; AUC= 0.71 [0.63 to 0.80]), but not with IDSA severity (*P = *0.51; AUC= 0.53 [0.45 to 0.61]), and similarly performed worse than our biomarker-based models (Fig. 2B).

The best biomarker-based elastic net model is able to improve the correct classification of 30-day all-cause mortality cases at time of diagnosis compared to the IDSA severity model for predicting 30-day all-cause mortality. This is demonstrated by a positive continuous net reclassification improvement (NRI) (*P = *0.022; NRI = 0.53 [0.078 to 0.98]) when comparing the two models. NRI ranges from −2 (100% of positives and 100% of negatives incorrectly reclassified) to +2 (100% of positives and 100% of negatives correctly reclassified); thus, an NRI of 0.53 is a moderate improvement in classification of individuals with 30-day all-cause mortality by the biomarker-basedmodel over the baseline IDSA severity model.

To test if Elixhauser and IDSA severity would add additional information to the models, we incorporated Elixhauser and IDSA severity into the best elastic net biomarker-based models (Fig. 2C and D) and into the best logistic regression models (Fig. S5) for 30-day mortality and DRCs. For alpha values of 0.9, the AUC for the 1se model for 30-day mortality increased from 0.89 (0.84 to 0.95) to 0.91 (0.84 to 0.97), while the AUC for the 1se model for DRCs increased from 0.84 (0.74 to 0.95) to 0.87 (0.78 to 0.97) with the addition of IDSA severity and Elixhauser. The 1se model for DRCs did not include Elixhauser as a coefficient, indicating poor predictive capability of that variable, while the addition of Elixhauser and IDSA severity resulted in procalcitonin not being included in the 1se model for 30-day all-cause mortality.

10.1128/mBio.00180-20.8 FIG S5 Inclusion of Elixhauser score and IDSA severity gives only slight improvements to best elastic net biomarker models. (A) ROC for min lambda elastic net models with an alpha value of 0.9 for predicting 30-day mortality and DRCs with and without the inclusion of Elixhauser and IDSA severity in addition to serum biomarkers. (B and C) ROCs of the best biomarker-based logistic regression models with the addition of Elixhauser or Elixhauser plus IDSA severity for 30-day mortality (B) and DRCs (C). While the addition of Elixhauser and IDSA severity increases AUC for the DRC model, it is not by a substantial margin. For 30-day mortality, the inclusion of Elixhauser and IDSA severity makes minimal improvement to the AUC. Download FIG S5, EPS file, 2.1 MB. Copyright © 2020 Dieterle et al. 2020 Dieterle et al. This content is distributed under the terms of the Creative Commons Attribution 4.0 International license.

### Multivariable, predictive models for 30-day mortality and DRCs do predict outcomes in a murine model of severe and nonsevere CDI.

We and others have developed murine models of CDI in which experimentally infected animals will develop disease ranging from mild diarrhea to severe colitis. These murine models of CDI allow us to test our predictive biomarker models in a model organism that can develop similar disease but lacks the potential comorbidities of human patients. We felt that this was important to assess, since if our models perform well in an animal system, this gives support to the notion that our models are being fit toward biologically relevant biomarkers for CDI rather than comorbid disease or other confounding features that would not be present in an animal system.

**FIG 3:**
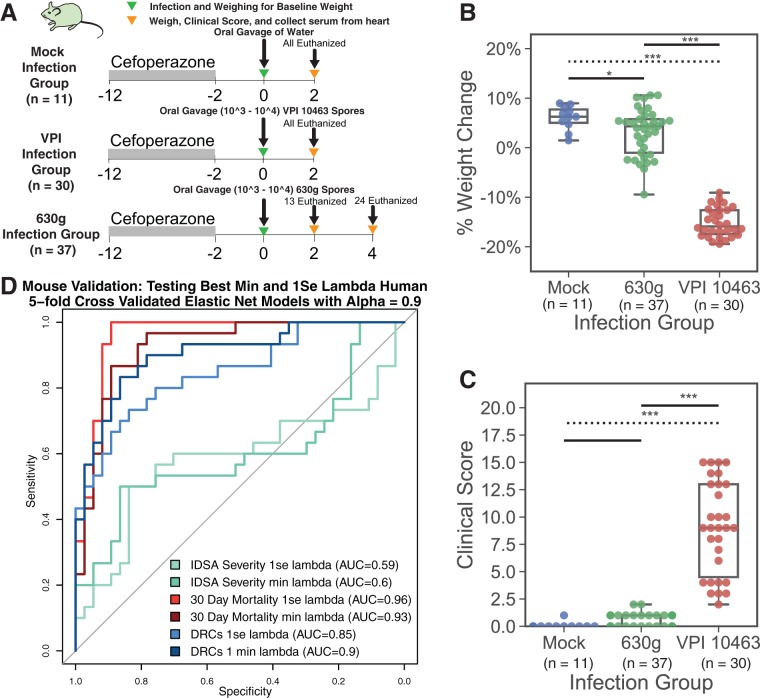
Mouse model of CDI to validates human CDI biomarker models. (A) Diagram showing method for mouse model of CDI. (B) Scatterplot showing weight change at day of euthanization compared to weight at day 0 for mock-infected, 630g-infected, and VPI 10463-infected mice. (C) Scatterplot showing clinical score (based on activity, coat, posture, diarrhea, and eyes/nose) at euthanization for mock-infected, 630g-infected, and VPI 10463-infected mice. VPI 10463-infected mice had more weight loss and higher clinical scores than 630g-infected mice (D). Mice infected with VPI 10463 were categorized as severe and those infected with 630g were categorized as nonsevere CDI cases. The 1se and min lambda elastic net models with alpha values of 0.9 for IDSA, 30-day mortality, and DRCs were applied to the mouse cohort with the resulting ROCs and AUCs. Weight change was analyzed using a *t* test with a Bonferroni *post hoc* adjustment, and clinical scores were analyzed using a Mann-Whitney U test with a Bonferroni *post hoc* adjustment. *, *P* < 0.05; **, *P* < 0.01; ***, *P* < 0.001.

For this validation, we used a CDI model employing antibiotic pretreatment followed by experimental infection with C. difficile spores. We have previously demonstrated that murine infection with VPI 10463 results in severe, rapidly fatal disease within 48 h, while infection with strain 630 results in a more indolent course (13). For these experiments we employed 78 antibiotic-treated mice that were challenged with either strain 630g (37 mice), strain VPI 10463 (30 mice), or water (11 mice) and assessed serum responses with a murine version of our multiplex panel. VPI 10463-infected mice exhibited higher weight loss, more histopathologic intestinal damage, and higher clinical severity (Fig. 3A to C and Fig. S6). Therefore, we classified mice infected with VPI 10463 to have severe and fatal CDI, while those infected with 630g were classified to have mild and nonfatal CDI. The best models from our human cohort were applied to the mouse cohort (best 1se and min lambda models with alpha of 0.9 for IDSA severity, 30-day mortality, and DRCs). Descriptions of which biomarkers are included in each model are found in Fig. 1B and Fig. S3b. To apply the models to the mice serum data, each outcome (severity/mortality/DRCs) was defined as positive for VPI 10463-infected and negative for 630g-infected mice (Fig. 3D) or by a cutoff of weight loss, cecum histopathology score, or colon histopathology score, as higher weight loss or histopathology represents more severe disease in mice with CDI (Fig. S7).

10.1128/mBio.00180-20.9 FIG S6 Unadjusted PCA and histological damage in mouse model of CDI. (A and B) Inflammatory and disease severity PCA plot (A) and RDA analysis (B) for 630g-infected mice (0), categorized as having nonsevere CDI infection, and VPI 10463-infected mice (1), categorized as having severe CDI infection. (C and D) Scatterplots showing cecum score (C) and colon score (D) based on the total score of edema (0 to 4), inflammatory cell infiltration (0 to 4), and epithelial damage (0 to 4) at euthanization for mock-infected, 630g-infected, and VPI 10463-infected mice. VPI 10463-infected mice had higher scores for cecum and colon damage than 630g-infected mice. Cecum and colon scores were analyzed using a Mann-Whitney U test with a Bonferroni *post hoc* adjustment. *, *P* < 0.05; **, *P* < 0.01; ***, *P* < 0.001. Download FIG S6, EPS file, 2.6 MB. Copyright © 2020 Dieterle et al. 2020 Dieterle et al. This content is distributed under the terms of the Creative Commons Attribution 4.0 International license.

10.1128/mBio.00180-20.10 FIG S7 ROCs for best 1se lambda and alpha 0.9 models produced on human validation cohort applied to mouse cohort. Severity for each model was defined by a cutoff in weight loss, cecum score, or colon score. The top panels show results for the IDSA model, the middle panels show results from the 30-day mortality model, and the bottom panels show DRC model results. (Left) ROC for defining severity by weight change from day 0 to euthanization, ranging from <1% to <14%. (Middle) ROC for severity defined as cecum scores of ≥4, 6, 8, or 10. (Right) ROC for severity defined as colon score ≥2, 4, 6, or 8. Cutoffs were chosen to range around median and mean of each metric. Overall, AUCs were stable across severity definitions. Download FIG S7, EPS file, 2.4 MB. Copyright © 2020 Dieterle et al. 2020 Dieterle et al. This content is distributed under the terms of the Creative Commons Attribution 4.0 International license.

The 1se models for prediction of 30-day all-cause mortality and DRCs accurately identified mice infected with high-virulence C. difficile. Specifically, applying each 1se model to the mouse cohort for high- versus low-virulence infections revealed AUCs of 0.59 (0.44 to 0.74) for the models built for IDSA severity, 0.96 (0.91 to 1.0) for the models built for 30-day mortality, and 0.85 (0.75 to 0.94) for the models built for DRCs.

## DISCUSSION

CDI is associated with an increased risk of mortality, and at present, we are inadequately determining who will experience adverse outcomes. Multiple models have been produced to address this problem, including those utilizing electronic medical records, standard laboratory tests, and medical history (6–12). However, these models have met with limited success in external validation, and there is room for improvement in predictive ability of CDI adverse outcomes. Additional studies have examined specific biomarkers in serum that could be associated with severe CDI, but no study to date has looked across a wide spectrum of serum-based biomarkers to determine their effectiveness of predicting cases of mortality or DRCs. Our results support the hypothesis that models built from a panel of multiple inflammatory mediators measured early in the course of CDI can accurately predict adverse outcomes and can do so better than current measures commonly used to predict adverse outcomes upon CDI diagnosis.

Our panel and model could be utilized at the time of diagnosis to evaluate the risk of mortality for an individual patient. A negative result reduces the risk of 30-day all-cause mortality, while a positive result increases mortality risk from ∼10% at baseline to ∼25%. Currently, the therapeutic options are limited in scope, but identifying a high-risk patient could tip the scale toward using more aggressive therapy, such as colectomy. A secondary use of the panel could be to enable the study of therapies targeted specifically at reducing mortality in CDI, which otherwise are infeasible due to lack of statistical power. For example, if a study was being performed for a therapy against standard of care with a theoretical 30% reduction of mortality in the standard population with baseline ∼10% mortality risk with a targeted power of 80% and an alpha (i.e., type I error) of 0.05, 2,700 patients would be required for the study. However, if our panel and model were used to identify only high-risk individuals that would be considered for enrollment, the population mortality risk would be increased to ∼25%, reducing the needed number of patients to 928. This would decrease the number of required subjects 3-fold, substantially reducing cost and improving feasibility.

Validation is an important step in determining if a model is overfit to the particular cohort and/or confounding factors rather than the disease process itself. Utilizing murine CDI allowed us to test the models in a separate system without potentially confounding factors such as age, treatments, and comorbidities. Our results show that the risk model of 30-day all-cause mortality and DRCs are related to the underlying biology of the infection as the models are also predictive of severe outcomes in murine CDI. Additionally, this provides additional support for the observation that murine CDI has a similar immune response to human CDI, supporting continued use of the animal model in the study of the biology of CDI.

Overall, our results confirm our hypothesis that a serum-based biomarker panel predicts adverse outcomes from CDI. Additionally, we show that models constructed from serum biomarkers outperform both IDSA severity criteria and Elixhauser comorbidity index for predicting adverse outcomes. Therefore, serum biomarker-based models could be used to inform medical decision-making for patients with CDI, and this study has explored models from a range of modeling algorithms to inform which biomarkers are the most promising. Specific interest should be placed on continuing to study HGF, procalcitonin, IL-8, IL-2Rα, IP-10, and CXCL-5, as they were the most prevalent biomarkers selected in models of adverse outcomes from CDI in this study.

## MATERIALS AND METHODS

### Cohort design.

Sera were collected within 48 h of diagnosis of CDI in two distinct cohorts of patients and frozen at −80°C until analysis. The pilot cohort collections ranged from October 2010 to November 2012, contemporaneous with our prior publications on various biomarkers in CDI. The validation cohort collections ranged from January to September 2016. We felt that it was important to separate these two cohorts as they were heterogeneous for several reasons: (i) the 4-year gap in time, (ii) the change in testing practices (e.g., collection of stool in Cary-Blair medium and no rejection of formed specimens in the pilot cohort era and use of best-practice alerts and educational alerts to modify testing protocols in the validation cohort era), and (iii) the change in treatment practices (movement away from metronidazole and toward fidaxomicin and vancomycin per new institutional guidelines). All patients were diagnosed with CDI by the clinical microbiology laboratory using a two-step algorithm including detection of C. difficile glutamate dehydrogenase (GDH) and toxins A and B by enzyme immunoassay (C. DIFF Quik Chek Complete; Alere, Waltham, MA), with reflex to PCR for *tcdB* gene for discordant results (Focus Simplexa assay from DiaSorin, Saluggia, Italy [pilot cohort], and BD Geneohm assay from Becton, Dickinson and Company, Franklin Lakes, NJ [validation cohort]). We examined prediction tasks for three major outcomes of interest: Infectious Disease Society of America (IDSA) severity, 30-day all-cause mortality, and disease-related complications (DRCs). An IDSA severe case is defined as leukocytosis with a white blood cell count of ≥15,000/ml or a serum creatinine level of >1.5 mg/dl (5). DRCs included colectomy, death, or ICU admission within 30 days attributed to CDI as determined by infectious disease (ID) physicians on our team blinded to the biomarker results (D.A.P. and K.R.).

The pilot cohort was analyzed with a 14-plex assay to examine key serum biomarkers. After our preliminary results and further study, CXCL-5, IL-22, and IL-23 were added to the panel to produce the 17-plex assay that was utilized on our validation cohort (Table 1). A similar panel was produced for mice with identical inflammatory mediators or the closest homologues. Our analysis was split into classification of current disease using IDSA severity as the gold standard and outcome prediction. The two outcomes of each CDI case that we set out to model were 30-day all-cause mortality from time of diagnosis and disease-related complications (DRCs), which included ICU admission, colectomy, or death caused by CDI specifically. Attributable CDI severity was determined through physician-based chart review.

This study was approved by the University of Michigan institutional review board (IRB).

### Human and mouse Luminex 17-plex assay.

Two custom, bead-based, multiplex inflammatory mediator panels were performed on samples using a Luminex 200TM dual-laser detection system. Our panel was selected from previous research cited in Table 1. The human multiplex panel included 17 inflammatory mediators previously identified as being potential biomarkers for CDI, including CCL-2 (MCP-1), CCL-4 (MIP-1b), CCL-5 (RANTES), CXCL-5, CXCL-9, CXCL-10 (IP-10), epidermal growth factor (EGF), hepatocyte growth factor (HGF), IL-2RΑ, IL-4, IL-6, IL-8, IL-15, IL-22, IL-23, procalcitonin (PCT), and TNF-α. The mouse 17-inflammatory-mediator panel included the same cytokines for murine serum except that it included KC, a mouse homologue of IL-8, instead of IL-8 and included LIX, a mouse homologue of CXCL-5, instead of CXCL-5. All resulting measurements, in picograms per milliliter, were log transformed. Demographic information and clinical variables were extracted from the electronic medical record for the human cohort.

### Data analysis methodology.

Given that measurements from Luminex assays are linear and thus accurate over a wide range of concentrations, generally spanning several orders of magnitude, the inflammatory mediator measurements were log transformed prior to analysis to correct for nonnormal distributions (positive skew). Principal-component analysis (PCA) was performed for the panel of inflammatory mediators, independent of our outcomes of interest, using princomp in the stats package in R (14). We performed redundancy analysis (RDA) for each binary variable (IDSA severity, 30-day all-cause mortality, and DRCs) as the predictor, and the outcomes were the log-transformed inflammatory mediators to assess whether the biomarker profile might be different between the individuals positive for the binary metric tested (e.g., those that experienced DRCs and those that did not). This was achieved by performing analysis of variance using Euclidean distance and a permutation test to find *P* values. This was performed using the vegan package in R (15). We assessed the impact of individual inflammatory mediators on the outcomes by performing unadjusted logistic regression for each inflammatory mediator.

We first attempted to model our outcomes using multivariable logistic regression with binomial deviance as our error measure. However, our overall goal was to identify important inflammatory mediators and construct models in a manner that avoided overfitting and would be more likely to generalize to an external cohort. With this in mind, we utilized 5-fold cross-validated elastic net multivariable logistic regression with the goal of testing the impact of adjusting the stringency of inclusion criterion and tuning parameters. A lambda value was selected where deviance was within 1 standard error of the minimum (1se, more stringent) or at the minimum (min). Additionally, we swept through alpha values range from pure ridge regression (alpha = 0) to pure lasso regression (alpha = 1) to identify which biomarkers would be included under each condition. For each value of alpha tested, 100 iterations across different seeds were performed. All of these methods (regularized regression, cross-validation, evaluating different lambda values, and sweeping the alpha tuning parameter) were aimed at avoiding overfitting, and even though this results in models that do not perform as well, the resulting claims about model performance are more conservative and more likely to validate externally. This was performed using the Glmnet package in R (16). Comparison of elastic net models was performed by creating receiver operating characteristic (ROC) curves and calculating the area under the ROC (AUC) using the pROC package in R (17). Net reclassification improvement index analysis was done using the PredictABEL package in R (18). All analysis was performed using R (19) and RStudio (20).

### Mouse experimental methods.

Eight- to 12-week-old, specific-pathogen-free (SPF) C57BL/6 mice were treated with 10 days of cefoperazone (0.5 g/liter) delivered in their drinking water to render them sensitive to Clostridioides difficile infection. The C57BL/6 mice used were produced by the Young Lab breeding colony at the University of Michigan established from mice purchased from the Jackson laboratory. After 2 days off antibiotics, mice were given an oral gavage of water, C. difficile 630g spores, or C. difficile VPI 10463 spores (Fig. 3). Inoculum was estimated between 10^3^ and 10^4^ spores. While mock-infected mice gained weight over the course of the observational time, 630g-infected mice remained at the same weight while VPI 10463-infected mice lost significant weight over 2 days. In our model, VPI 10463 infection following cefoperazone will result in a high proportion of death if allowed to progress beyond 48 h. To obtain serum samples, VPI 10463-infected mice were sacrificed 2 days after infection, while half of the 630g-infected mice were sacrificed at day 2 as time controls and the rest were sacrificed at 4 days postinfection, when they reached their maximum disease. Cecum and colon histopathology were scored from 0 to 12 by a blinded pathologist for edema, epithelial damage, and inflammatory cell infiltration. Each mouse was given a clinical score from 0 to 20 at euthanization based on posture, coat, activity, diarrheal signs, and weight change from day 0 (D0). Further description of the model can be found in the work of Leslie et al. 2019 (33).
