# Supplementary material for: Systemic Inflammatory Mediators Are Effective Biomarkers for Predicting Adverse Outcomes in Clostridioides difficile Infection
Source: mBio. 2020 May 5;11(3):e00180-20. doi: 10.1128/mBio.00180-20 (PMC7403776; doi:10.1128/mBio.00180-20)
Supplement: TABLE S1 [file mBio.00180-20-st001.docx]

| **Table S1: Pilot Cohort -- Biomarker Population Statistics and Simple Unadjusted Logistic Regression Analysis for IDSA Severity and the Disease Outcomes 30-Day-All-Cause Mortality and Disease Related Complications (DRCs)** | | | | | | | | | | | | |
| --- | --- | --- | --- | --- | --- | --- | --- | --- | --- | --- | --- | --- |
| **Individual Log Transformed Biomarker Population Statistics** | | | | **Unadjusted Analysis for IDSA Severity** | | | **Unadjusted Analysis for 30 Day Mortality** | | | **Unadjusted Analysis for DRC** | | |
| **Bio-marker** | **Median** | **Mean** | **Std.** | **Bio-marker** | **Odds Ratio** | **Sign.** | **Bio-marker** | **Odds Ratio** | **Sign.** | **Bio-marker** | **Odds Ratio** | **Sign.** |
| IL-6 | 3.18 | 3.24 | 1.42 | IL-6 | 1.71 [1.30-2.24] | *** | IP-10 | 2.33 [1.09-4.96] | * | HGF | 2.07 [1.22-3.52] | ** |
| PCT | 0.11 | 0.46 | 0.90 | PCT | 2.95 [1.53-5.70] | ** | IL-6 | 2.05 [0.98-4.27] | - | IL-8 | 2.07 [1.16-3.68] | * |
| IL-8 | 3.47 | 3.44 | 1.02 | IL-8 | 1.45 [1.04-2.04] | * | IL-4 | 0.58 [0.31-1.09] | - | IL-2R | 1.84 [1.12-3.02] | * |
| IL-2R | 6.07 | 5.78 | 1.75 | IL-2R | 1.27 [1.02-1.58] | * | MCP-1 | 0.63 [0.28-1.41] | - | IL-6 | 1.76 [1.09-2.84] | * |
| HGF | 6.23 | 6.03 | 1.71 | HGF | 1.25 [1.00-1.57] | * | CXCL-9 | 0.57 [0.21-1.58] | - | TNFa | 0.52 [0.27-0.98] | * |
| EGF | 2.80 | 2.99 | 1.19 | EGF | 1.25 [0.91-1.70] | - | IL-8 | 1.56 [0.67-3.65] | - | MCP-1 | 2.06 [1.02-4.15] | * |
| RANTES | 8.58 | 8.46 | 1.00 | RANTES | 0.85 [0.60-1.19] | - | IL-2R | 0.90 [0.52-1.56] | - | MIP-1b | 1.44 [0.73-2.82] | - |
| IL-15 | 3.50 | 3.28 | 1.44 | IL-15 | 1.09 [0.85-1.40] | - | TNFa | 0.85 [0.33-2.21] | - | EGF | 1.32 [0.76-2.30] | - |
| MIP-1b | 4.70 | 4.70 | 0.98 | MIP-1b | 1.11 [0.77-1.9] | - | MIP-1b | 0.85 [0.31-2.33] | - | CXCL-9 | 1.22 [0.70-2.13] | - |
| IP-10 | 4.89 | 5.15 | 1.35 | IP-10 | 1.05 [0.81-1.36] | - | RANTES | 0.87 [0.32-2.35] | - | IL-4 | 0.87 [0.54-1.40] | - |
| IL-4 | 3.66 | 3.00 | 1.25 | IL-4 | 0.96 [0.74-1.24] | - | HGF | 0.93 [0.53-1.64] | - | PCT | 1.09 [0.53-2.24] | - |
| MCP-1 | 5.57 | 5.24 | 1.17 | MCP-1 | 0.99 [0.73-1.34] | - | PCT | 1.17 [0.33-4.12] | - | IL-15 | 1.02 [0.65-1.61] | - |
| CXCL-9 | 4.25 | 4.20 | 1.13 | CXCL-9 | 1.01 [0.74-1.39] | - | IL-15 | 0.94 [0.48-1.84] | - | IP-10 | 0.98 [0.61-1.58] | - |
| TNFa | 1.79 | 2.06 | 1.06 | TNFa | 1.00 [0.73-1.36] | - | EGF | 1.04 [0.45-2.43] | - | RANTES | 1.01 [0.53-1.92] | - |
