# Supplementary material for: Systemic Inflammatory Mediators Are Effective Biomarkers for Predicting Adverse Outcomes in Clostridioides difficile Infection
Source: mBio. 2020 May 5;11(3):e00180-20. doi: 10.1128/mBio.00180-20 (PMC7403776; doi:10.1128/mBio.00180-20)
Supplement: TABLE S2 [file mBio.00180-20-st002.docx]

| **Table S2: Validation Cohort -- Biomarker Population Statistics and Simple Unadjusted Logistic Regression for IDSA Severity and the Disease Outcomes 30 Day Mortality and Disease Related Complications (DRCs)** | | | | | | | | | | | | | | | |
| --- | --- | --- | --- | --- | --- | --- | --- | --- | --- | --- | --- | --- | --- | --- | --- |
| **Individual Log Transformed Biomarker Population Statistics** | | | | **Unadjusted Analysis for IDSA Severity** | | | | **Unadjusted Analysis for 30 Day Mortality** | | | | **Unadjusted Analysis for DRC** | | | |
| **Bio-marker** | **Median** | **Mean** | **Std.** | **Bio-marker** | **Odds Ratio** | **OR Sig.** | **AUC** | **Bio-marker** | **Odds Ratio** | **OR Sig.** | **AUC** | **Bio-marker** | **Odds Ratio** | **OR Sig.** | **AUC** |
| HGF | 5.85 | 5.95 | 1.06 | HGF | 1.97 [1.49-2.60] | *** | 0.71 [0.64-0.78] | IL-2Ra | 8.28 [3.41-20.11] | *** | 0.85 [0.77-0.92] | PCT | 1.94 [1.43-2.64] | *** | 0.82 [0.75-0.90] |
| PCT | 4.83 | 5.35 | 1.43 | PCT | 1.57 [1.3-1.89] | *** | 0.69 [0.62-0.76] | PCT | 1.92 [1.42-2.58] | *** | 0.82 [0.73-0.9] | IL-8 | 2.03 [1.44-2.86] | *** | 0.78 [0.67-0.88] |
| IL-6 | 2.68 | 3.02 | 1.57 | IL-6 | 1.39 [1.17-1.65] | *** | 0.68 [0.62-0.75] | IL-8 | 2.03 [1.45-2.86] | *** | 0.80 [0.71-0.90] | IL-2Ra | 4.86 [2.16-10.94] | *** | 0.79 [0.69-0.88] |
| IL-2Ra | 7.6 | 7.61 | 0.65 | IL-2Ra | 2.29 [1.47-3.57] | *** | 0.65 [0.58-0.72] | IP-10 | 1.76 [1.31-2.36] | *** | 0.67 [0.54-0.79] | IL-6 | 1.49 [1.17-1.90] | ** | 0.69 [0.54-0.84] |
| IL-8 | 3.65 | 3.88 | 1.16 | IL-8 | 1.44 [1.14-1.82] | ** | 0.64 [0.57-0.71] | EGF | 0.59 [0.43-0.8] | *** | 0.70 [0.58-0.83] | HGF | 1.94 [1.3-2.89] | ** | 0.71 [0.59-0.84] |
| TNFa | 4.01 | 3.89 | 0.51 | TNFa | 2.87 [1.29-6.39] | ** | 0.61 [0.53-0.69] | CXCL-5 | 0.53 [0.36-0.8] | ** | 0.75 [0.65-0.85] | IP-10 | 1.42 [1.05-1.94] | * | 0.62 [0.49-0.75] |
| MIP-1b | 6.16 | 6.1 | 0.4 | MIP-1b | 2.48 [1.16-5.30] | * | 0.61 [0.53-0.68] | IL-6 | 1.44 [1.13-1.83] | ** | 0.70 [0.55-0.84] | MCP-1 | 1.58 [0.98-2.56] | - | 0.62 [0.45-0.79] |
| IL-22 | 6.07 | 5.96 | 0.51 | IL-22 | 1.93 [1.03-3.63] | * | 0.59 [0.51-0.67] | RANTES | 0.7 [0.51-0.97] | * | 0.73 [0.61-0.85] | CXCL-5 | 0.71 [0.47-1.06] | - | 0.63 [0.51-0.75] |
| IL-15 | 3.06 | 2.79 | 1.14 | IL-15 | 1.23 [0.95-1.60] | - | 0.57 [0.49-0.65] | IL-22 | 0.45 [0.22-0.93] | * | 0.61 [0.45-0.76] | EGF | 0.77 [0.56-1.07] | - | 0.62 [0.48-0.76] |
| RANTES | 9.61 | 9.34 | 0.96 | RANTES | 1.3 [0.88-1.93] | - | 0.55 [0.48-0.63] | HGF | 1.53 [1.03-2.27] | * | 0.63 [0.47-0.78] | IL-15 | 1.39 [0.82-2.35] | - | 0.65 [0.50-0.80] |
| EGF | 4.14 | 3.89 | 1.32 | EGF | 1.14 [0.92-1.41] | - | 0.54 [0.47-0.62] | MCP-1 | 1.58 [0.98-2.53] | - | 0.62 [0.47-0.78] | RANTES | 0.84 [0.59-1.20] | - | 0.61 [0.48-0.75] |
| IL-4 | 5.25 | 5.1 | 0.48 | IL-4 | 1.3 [0.70-2.38] | - | 0.49 [0.41-0.57] | IL-4 | 0.52 [0.25-1.06] | - | 0.66 [0.52-0.80] | IL-4 | 0.78 [0.33-1.83] | - | 0.57 [0.42-0.72] |
| MCP-1 | 5.75 | 5.89 | 0.85 | MCP-1 | 1.14 [0.84-1.56] | - | 0.52 [0.44-0.60] | IL-15 | 1.7 [0.95-3.07] | - | 0.66 [0.52-0.80] | MIP-1b | 1.29 [0.38-4.43] | - | 0.56 [0.40-0.72] |
| CXCL-5 | 6.79 | 6.7 | 1.1 | CXCL-5 | 1.1 [0.85-1.41] | - | 0.52 [0.45-0.60] | MIP-1b | 0.41 [0.14-1.17] | - | 0.57 [0.41-0.74] | TNFa | 1.20 [0.42-3.40] | - | 0.51 [0.37-0.65] |
| CXCL-9 | 6 | 5.82 | 1.39 | CXCL-9 | 1.08 [0.87-1.33] | - | 0.58 [0.50-0.65] | CXCL-9 | 0.87 [0.66-1.13] | - | 0.52 [0.35-0.70] | IL-23 | 0.96 [0.65-1.42] | - | 0.58 [0.45-0.72] |
| IP-10 | 2.65 | 2.99 | 1.18 | IP-10 | 0.97 [0.77-1.23] | - | 0.55 [0.47-0.63] | TNFa | 0.71 [0.34-1.48] | - | 0.57 [0.42-0.72] | CXCL-9 | 1.01 [0.71-1.45] | - | 0.49 [0.34-0.65] |
| IL-23 | 3.75 | 3.48 | 1.25 | IL-23 | 0.98 [0.78-1.22] | - | 0.51 [0.43-0.59] | IL-23 | 0.87 [0.61-1.24] | - | 0.58 [0.43-0.72] | IL-22 | 0.97 [0.38-2.47] | - | 0.53 [0.39-0.66] |
