# Supplementary material for: Systemic Inflammatory Mediators Are Effective Biomarkers for Predicting Adverse Outcomes in Clostridioides difficile Infection
Source: mBio. 2020 May 5;11(3):e00180-20. doi: 10.1128/mBio.00180-20 (PMC7403776; doi:10.1128/mBio.00180-20)
Supplement: TABLE S3 [file mBio.00180-20-st003.docx]

| **Table S3 -- Logistic Regression with backward selection for estimating IDSA Severity and predicting adverse outcomes (30 Day Mortality and DRCs). The resulting AUC is stable for each outcome across biomarker inclusion and selection processes. mAIC = Stepwise Regression, Backward Selection with all biomarkers. m25AIC = Stepwise Regression, Backward Selection including only biomarkers with P < 0.25 from unadjusted logistic regression. mLRT = Stepwise Regression, Drop One Selection with all biomarkers. m25LRT = Stepwise Regression, Drop One Selection including only biomarkers with P < 0.25 from unadjusted logistic regression.** | | | | | |
| --- | --- | --- | --- | --- | --- |
| **Models Estimating IDSA Severity Models** | | | | | |
| **Model** | **Included Biomarkers** | **AIC** | **BIC** | **Log-Likelihood** | **AUC** |
| mAIC | HGF, Procalcitonin, RANTES, and CXCL-9 | 254.41 | 272.12 | -122.21 | 0.75  [0.68-0.82] |
| m25AIC | HGF, Procalcitonin, and EGF | 277.80 | 292.17 | -134.90 | 0.74  [0.68-0.81] |
| mLRT | HGF, Procalcitonin, and RANTES | 255.78 | 269.95 | -123.89 | 0.75  [0.68-0.82] |
| m25LRT | HGF and Procalcitonin | 279.51 | 290.28 | -136.75 | 0.73 [0.67-0.8] |
| **Models Predicting 30-DayAll-Cause Mortality** | | | | | |
| **Model** | **Included Biomarkers** | **AIC** | **BIC** | **Log-Likelihood** | **AUC** |
| mAIC | IL-2Ra, IL-8, CXCL-5, HGF, IP-10, IL-6, CXCL-9 and TNFa | 94.58 | 126.52 | -38.29 | 0.9  [0.82-0.98] |
| m25AIC | IL-2Ra, IL-8, CXCL-5, HGF, IP-10, IL-6 and IL-15 | 105.83 | 134.61 | -44.91 | 0.9  [0.83-0.97] |
| mLRT | IL-2Ra, IL-8, CXCL-5, HGF, IP-10 and IL-6 | 94.40 | 119.24 | -40.20 | 0.9  [0.82-0.97] |
| m25LRT | IL-2Ra, IL-8 and CXCL-5 | 108.23 | 122.62 | -50.11 | 0.89  [0.84-0.95] |
| **Models Predicting Disease Related Complications (DRCs)** | | | | | |
| **Model** | **Included Biomarkers** | **AIC** | **BIC** | **Log-Likelihood** | **AUC** |
| mAIC | IL-8, HGF, and IL-2Ra | 100.84 | 115.04 | -46.42 | 0.85  [0.74-0.96] |
| m25AIC | IL-8, HGF, and IL-2Ra | 111.78 | 126.21 | -51.89 | 0.85  [0.75-0.94] |
| mLRT | IL-8, HGF, and IL-2Ra | 100.84 | 115.04 | -46.42 | 0.85  [0.74-0.96] |
| m25LRT | IL-8, HGF, and IL-2Ra | 111.78 | 126.21 | -51.89 | 0.85  [0.75-0.94] |
